# Nutritional Dermatology: Optimizing Dietary Choices for Skin Health

**DOI:** 10.3390/nu17010060

**Published:** 2024-12-27

**Authors:** Sandi Assaf, Owen Kelly

**Affiliations:** Department of Molecular and Cellular Biology, College of Osteopathic Medicine, Sam Houston State University, Conroe, TX 77304, USA; ojk003@shsu.edu

**Keywords:** skin health, UV rays, phytochemicals, dietary antioxidants, dermatology

## Abstract

Background/Objectives: Youthful, smooth skin is highly desired in modern society. Individuals invest in cosmetics, plastic surgeons, and dermatologists in pursuit of perfect skin. However, many do not seek out dietary changes to improve skin health. Although research has been conducted on the role of nutrition and select nutrients and phytonutrients on skin health, there is a lack of healthy food recommendations for clear skin. Methods: The literature was assessed to determine which nutrients and phytonutrients play a significant role in the protection and maintenance of skin health. Key compounds were highlighted as there is evidence to suggest they have a significant role in skin health: vitamin A, vitamin C, vitamin D, vitamin E, zinc, omega-6 and omega-3 fatty acids, polyphenols/flavonoids, copper, selenium, and silicon. USDA FoodData Central and FooDB (food database), were utilized to select foods and food groups containing the key nutrients and phytonutrients. Results: A skin-healthy dietary pattern is proposed in addition to a scoring system to assess diet. A sample skin-healthy daily diet was designed, using only whole foods, that met the Daily Values for vitamins and minerals and contained key compounds for skin health. Conclusions: There is a clear link between nutrition and skin health, or nutritional dermatology; however, more research needs to be done to find the intersection between both disciplines.

## 1. Introduction

Youthful, homogenous, healthy-looking skin has remained desirable among cultures despite changes to the definition of beauty [1]. The size of the skin care industry shows how skin health is important in modern society. Skin care products, in 2024 globally, have an estimated value of USD 149.4 billion and a forecasted yearly growth over the next seven years [2], and skin health supplements alone were valued at USD 12 billion in 2023 [3]. A report from the American Society of Plastic Surgeons adds another perspective. In 2023 there were over 25 million minimally invasive procedures, a 7% increase over 2022, and a 15% growth for men [4]. Minimally invasive procedures include neuromodulator injections, skin resurfacing, and hyaluronic acid fillers. To give an idea of the spending on minimally invasive procedures, there were over nine million neuromodulator injections with an average cost of USD 435.

Skin cancers are a major concern and frequent use of sunscreen is the default advice to protect against them—prevention is better than a cure. Medicare data from 2013 show that USD 2.5 billion was spent on skin cancer, with half of the spending related to basal cell carcinoma [5]. This figure is likely much higher for the whole U.S. population as it does not include any data from people younger than 65 years. Sunscreen in general protects the skin from harmful UV-B rays (some block both UV-B and UV-B), while sunblock stops a broad spectrum of UV light. However, chemical sunscreens contain ingredients such as oxybenzone, octocrylene, and octisalate, which remain in the plasma and can reach potentially toxic concentrations [6]. Other ingredients in sunscreens such as parabens have been found to interfere with endocrine pathways, especially human epidermal growth factor receptor 2 (HER2), via interacting with other HER receptors, and may stimulate breast cancer cell proliferation in vitro [7,8]. Overall, while the advice to block UV light can help reduce skin cancers, chemicals from sunscreens do enter the blood and there must be a balanced frequency of use until robust safety data are available [9]. In addition, other options need to be pursued, making nutritional dermatology a viable adjunctive therapy.

The American Academy of Dermatology’s clinical guidelines include dietary advice for the management of acne (avoid high-glycemic foods) [10], atopic dermatitis (elimination diets, essential fatty acids) [11], basal cell carcinoma (do not supplement with selenium or β-carotene) [12], cutaneous squamous cell carcinoma (do not supplement with selenium or β-carotene) [13] and psoriasis (various dietary supplements, although listed as alternative medicine) [14]. However, no dietary recommendations were included for actinic keratosis, melanoma, and reconstruction after skin cancer resection. Dietary guidelines are expected to be included for the latter two as diet is important for cancer prevention, during cancer treatment, and for wound healing. Alternatives, such as the Dermatology Medical Nutrition Therapy recommendations [15], the role of the Western diet in skin inflammation [16], and the RACGP aged care clinical guide (Silver Book), recommend a healthy diet to maintain skin integrity and prevent damage [17]; these current recommendations may help in the creation of future guidelines.

Skin care products are advertised with their value and potency based on the presence of various micronutrients that have been shown to have a structure–function relationship with skin health. This shows that people are already aware of the potential of nutritional dermatology. Topical applications may have limited value depending on whether the nutrient (or compound) is well absorbed through the skin, whether it has local or systemic effects, and whether it has acute or longer term effects. It must be noted that although skin and the gastrointestinal system share outward-facing epithelial cells, the skin was not designed to absorb nutrients; this is a feature unique to the gastrointestinal system. As recently reviewed, a good dietary pattern (predominately whole foods) is associated with better skin health [18,19,20] and differences between male and female skin health probably exist [21]. It is unknown if the consumption of processed foods supplemented with phytonutrients can influence the skin in a similar way.

The gut-skin axis [22], a relatively new concept, has already been extensively reviewed, see [22,23,24,25]. Briefly, when food is ingested, the resident microbes utilize some of the nutrients and other food compounds, including phytonutrients, to produce new metabolites which may act as systemic messenger molecules for the cutaneous microbiota [16]. Thus, a poor diet may contribute to dysbiosis, inflammation, and reduced skin health, resulting in skin conditions. Furthermore, treating skin disorders with antibiotics may also contribute to dysbiosis [26]. Skin, like the gastrointestinal system, also has its own microbiome. While inherently complex, and with variations in the microbial composition depending on the skin site, one study showed that the microbiome composition was relatively stable over a two-year period [27]. Reductions in *Lactobacillus* and *Cutibacterium* occur with age [28], and cutaneous dysbiosis probably occurs when skin conditions are present [29,30,31], allowing the possibility for future therapies [32]. However, it is unknown how different dietary patterns affect the skin microbiome. Moreover, protection from UV through dietary components, either directly as topical applications or indirectly through a diet enriched by phytonutrients, is also being explored [33].

The skin and gastrointestinal systems can be negatively influenced by a plethora of factors such as chemicals from pollution or processed foods, as was studied in athletic individuals [34]. When severe, this dysbiosis and immune dysregulation may manifest as cutaneous diseases such as acne, atopic dermatitis, and psoriasis. These pathophysiologies may be alleviated by dietary changes, prebiotics, and probiotics [35]. This suggests that encouraging the consumption of whole foods with abundant phytonutrients would maintain a healthy gut and cutaneous microbiomes [36]. It is essential to recognize that topical treatment of skin with phytonutrients (referred to as cosmeceutics) has also been shown to positively influence the scalp and the skin microbiota, e.g., *Vitis vinifera* L. leaves [37].

There is ample evidence to support the role of a good diet, especially plant-based whole foods, in promoting or maintaining skin health [18,20,38,39,40,41,42]. Furthermore, the American Academy of Dermatology includes nutritional guidelines for some disorders in clinical practice. In the U.S., between 2007 and 2018, there were a total estimated 1.55 billion visits to the dermatologist for a range of skin disorders [43]. Skin health is important to individuals as evidenced by the size of the skin care industry and the number of minimally invasive procedures performed during plastic surgeon and dermatology visits. Nutrition plays a role in many fields of medicine, especially cardiology, where diet is intrinsically linked with the risk of cardiovascular disease. This has resulted in the widely recognized concept of “heart-healthy foods” [44]. However, the same level of integration has not occurred in dermatology. Although ongoing research into the role of specific dietary compounds in skin health is important to generate evidence, a significant gap related to the translation of the research for public health still exists. The renewed interest in Nutrition as Medicine (or Food is Medicine) [45] also adds momentum to the need for more practical applications of nutrition research. Therefore, this review aims to briefly summarize current knowledge and apply this knowledge to propose a preliminary skin-healthy diet to help bring nutritional dermatology into daily practice.

## 2. Nutrients for Skin Health

Diet is important to maintain skin health [46]. A review of the current literature resulted in the following list of the most cited nutrients and compounds related to skin health: vitamin A, carotenoids, vitamin C, vitamin D, vitamin E, zinc, copper, selenium, silicon, polyphenols, and essential fatty acids. A review of all the mechanisms involved for each of these is beyond the scope of this review; in addition, these compounds have been the topic of several recent reviews. Therefore, the role of these nutrients in skin health will be briefly summarized before translating these findings into a diet for skin health. While the list seems small, carotenoids consist of numerous compounds, although few have been extensively researched (β-carotene, lycopene, astaxanthin).

### 2.1. Vitamin A and Carotenoids

Vitamin A plays a role in preventing disease and microbial infections of the skin, whereas deficiency is associated with an increase in the former [47]. All-trans-retinoic acid has been shown to be effective when applied topically for mild acne, while deficiency has been linked to delayed wound healing, and atopic dermatitis is associated with lower vitamin A status and dysfunctional retinoid signaling [48]. Retinoids have also been shown to be effective in reducing the appearance of skin aging and discoloration [46]. The plant-based carotenoid β-carotene has been shown to reduce erythema from UV [49]. β-carotene and other carotenoids (lycopene, lutein, zeaxanthin) from plants can be found on the skin and follow seasonal variation. Furthermore, carotenoids can be measured on the skin and may act as protection for the skin [50]. Lycopene, found in red fruits and vegetables, has also been shown to decrease oxidative damage in the skin [49]. Lycopene is negatively correlated with skin roughness due to its role as the most effective carotenoid singlet neutralizer [33]. Parsley was shown to have the highest concentration of carotenoids across several herbs [51]. However, astaxanthin, found in marine life, also conveys UV protection [49]. Regular consumption of astaxanthin was also found to reduce the aging of residual skin surface components, as well as provide photoprotection [47]. The mechanism of action may work by inhibiting the formation of reactive oxygen species and increasing the expression of enzymes that respond to oxidative damage [52]. Although there is a dietary recommended intake for Vitamin A, there are no requirements for carotenoids.

### 2.2. Vitamin C

Vitamin C is critical for skin health because of its role in stimulating collagen and elastin synthesis and inhibiting melanin production. Vitamin C also protects against UV-A and UV-B by inhibiting proinflammatory cytokines and apoptosis; it also has the potential to protect against several skin diseases such as atopic dermatitis, herpes, and malignant melanoma, though its mechanism as a treatment is not well understood [46]. Although skin care products include vitamin C, it is not as effective as the systemic route, as vitamin C degrades quickly when oxidized and loses its reducing capacity which carries its function [49]. Vitamin C also improves hydration in the epidermis [48].

### 2.3. Vitamin D

Vitamin D is synthesized from 7-dehydro-cholesterol in the skin. Vitamin D deficiency may exacerbate atopic dermatitis while supplementation has been found to be effective in treating patients with psoriasis due to its regulation of cAMP and ability to decrease inflammation and improve wound healing [48]. Vitamin D has an important role in preventing UV-mediated damage to cells and preventing infections. However, a person’s ability to synthesize vitamin D from sunlight decreases with age which can lead to increased sun damage and infections [49]. Active vitamin D seems protective against UV-B DNA damage in pretreated keratinocytes in vitro [53]. Nevertheless, it may be both the synthesis of vitamin D (absorb UV) and active vitamin D that have protective effects against UV [53,54].

### 2.4. Vitamin E

Vitamin E (α-tocopherol) protects against oxidative stress in lipids and therefore can be supplemented in patients who have psoriasis and atopic dermatitis [47]. Vitamin E is protective against collagen breakdown in the skin and decreases skin inflammation, while deficiency has been linked to irregular collagen structure and the presence of skin ulcers [48]. This vitamin has a historic role in dermatology and has been shown to be effective in delaying the growth of skin cancer, improving hyperpigmentation, and enhancing the integrity of epidermal and dermal structures for the delay of skin aging [46].

### 2.5. Fatty Acids

Fatty acids are a group of at least 20 compounds differentiated by the number of carbons and double bonds [55]. They are used in cosmetics; however, they have a very important role in maintaining the skin barrier, pH, and moisture level [56]. Linoleic acid (ω-6) and α-linolenic acid (ω-3) are the essential fatty acids. Other important ω-6 fatty acids are gamma-linolenic acid (GLA) and arachidonic acid (AA), and important ω-3 are eicosapentaenoic acid (EPA) and docosahexaenoic acid (DHA). The presence of these fatty acids has been shown to relieve skin inflammation and dryness [47]. More importantly, epidermal tissue requires an adequate supply of essential fatty acids for homeostasis [56]. ω-3 fatty acid supplementation has been shown to be effective, particularly in patients with psoriasis and atopic dermatitis [57]. Additionally, 1.8 g/d of EPA and 1.2 g/d of DHA have been shown to reduce erythema, while 4 g/d of EPA has been shown to reduce UV-induced irradiation and may even reduce the risk of skin cancer. Linoleic deficiency has been linked to skin dryness due to its role in the skin barrier while deficiency of GLA is linked to excessive epidermal exfoliation [46]. Inversely, increased linoleic acid consumption is negatively correlated with skin dryness and atrophy [49], suggesting there needs to be a low ratio of ω-6 to ω-3 fatty acids. There is also some evidence to suggest that a low ratio of ω-6 to ω-3 fatty acids is required for the treatment of atopic dermatitis, psoriasis, and acne [58]. Foods rich in EFAs have a therapeutic effect on damaged skin, especially dehydration, and must be considered beneficial for skin health [46]. Skin is a barrier against infection and the cutaneous microbiota produces short-chain fatty acids which have antibacterial effects, directly by decreasing local pH and indirectly by immune system activation [56]. Of all the fatty acids, the long chain ω-3 seem to have the most potential as adjunctive therapy for preventing and treating skin conditions, possibly due to their inflammatory mediating properties. Supplemental ω-3 fatty acids are relatively safe (few side effects) and have shown benefits for photoprotection, psoriasis, eczema, retinoid-induced cutaneous side effects, and during chemotherapy, see reviews [57,59]. As a final point, fats are required for the absorption of dietary fat-soluble nutrients and phytonutrients.

### 2.6. Polyphenols

Polyphenols is the umbrella term for at least 8,000 compounds (phytonutrients) that are ubiquitous in plants; for reference, a glass of wine or coffee can contain 100 mg of polyphenols [60]. Polyphenols inhibit enzymes that degrade collagen and elastin, thus improving skin quality and maintaining the skin’s structural integrity. Polyphenols also have antimicrobial properties, can reduce oxidative damage to the skin, and have been shown to prevent wrinkle formation [46]. Polyphenols can be further classified into five main groups: flavonoids (e.g., quercetin), phenolic acids (e.g., caffeic acid), stilbenes (e.g., resveratrol), tannins (e.g., ellagitannins), and diferuloylmethane (e.g., curcumin) [46], with flavonoids being the largest group [61]). Flavonoids are further classified into flavones, flavanols, isoflavones, and flavanones, showing the diversity in compounds. Quercetin is a polyphenolic flavonoid found in several foods, which has a yellow color and is widely known for its array of benefits in diseases from diabetes to cancer [62]. However, quercetin also has a major role in skin health. Evidence suggests that quercetin protects against photoaging and can be used topically to maintain glutathione levels in the skin, improve wound healing, and help reduce scarring, along with its antibacterial properties; see reviews by Zaborowski et al. [63] and Aghababaei and Hadidi [64]. Quercetin has also been shown to downregulate inflammatory cytokines associated with atopic dermatitis such as interleukin-4 and -5 [65]. Other flavonoids have similar effects on the skin, see review [66], as well as in positively modulating the intestinal microbiome [67]. The flavanol kaempferol has been shown to prevent dermal fibroblastic apoptosis and the formation of reactive oxygen species, thereby inhibiting the inflammatory cascade [68]. Cocoa has been shown to have one of the highest concentrations of antioxidants and flavonoids, with protective effects against skin aging, oxidative damage, and carcinogenesis [69]. Consuming flavanol-rich cocoa was also shown to cause a short-term increase in oxygen saturation and blood flow in the dermis [70], which may contribute to healthier skin.

Work from 1983 showed that dietary plant sterols are transported in plasma to the skin and surface lipids [71], and as dietary intake decreases, skin and fecal concentrations decrease. This followed earlier similar work [72] that showed cholesterol is important for skin health, which may help explain the skin-related side effects of statins [73]. This suggests that a constant daily intake of phytonutrients and dietary fat is required to maintain skin phytochemical concentrations. Supplementation with aloe vera sterol-containing gel powder decreased facial wrinkles, possibly by increasing hydration, when supplemented to Japanese women with dry skin for 8 weeks [74]. The authors confirmed that dietary aloe vera sterols reached the skin. Cholesterol and phytosterols are important in skin health, see review [75], and may have antiaging properties [76]. A lower-dose aloe vera sterol-containing gel powder (19 µg/d), increased skin barrier function and hydration in healthy adult females [77]. Other evidence shows that topical phytosterols relieve methyl nicotinate-induced erythema and maintain collagen synthesis after UV exposure [78]. Maintaining youthful skin is important for many people, and while it may seem that supplementation with pharmacological doses of polyphenols and nutrients is the best strategy, it may be best to consume a diet containing whole fruits and vegetables so polyphenols are at physiological doses [49,79] It is likely that many other plant polyphenols have skin benefits; however, more research is needed to elucidate which polyphenols have the greatest benefit.

### 2.7. Zinc

Zinc is a mineral and cofactor for various metalloenzymes. The role of zinc in skin health has been reviewed [46,80,81]. Zinc deficiency causes skin disorders, including dermatitis due to zinc’s important role in maintaining skin homeostasis [82]. Zinc is concentrated in the epidermis and has an anti-inflammatory role, as well as modulating apoptosis [80]. Zinc also has wound healing and microbial properties, and so has been used topically [81]. Zinc oxide is the main ingredient in mineral-based sunblock and functions by absorbing UV rays before they penetrate the skin; it also synergistically works to prevent infections with other micronutrients [48]. In addition to sun protection, zinc has been shown to be effective in treating inflammatory dermatoses and pigmentation disorders, as well as improving conditions such as actinic keratoses and alopecia areata [46], and wound healing [83].

### 2.8. Copper

Copper is a micronutrient and can protect the skin from UV damage. Copper also allows for collagen maturation and melanin synthesis (7). Copper has antimicrobial properties and copper peptides (glycyl-histidyl-lysine (GHK) or γ-glutamyl-cysteinyl-glycine (GSH)) have been shown to regenerate skin tissue and decrease oxidative damage in the skin [46]. It specifically increases the expression and binding of hypoxia-inducible factor 1-alpha (HIF-1α) for angiogenesis and induces vascular endothelial growth factor (VEGF) for the regeneration of new skin in wound healing [84]. These properties have made it an important component in wound dressings. Additionally, males with acne vulgaris have a lower Zn–Cu ratio along with dependent antioxidant enzymes which may indicate therapeutic potential for copper supplementation [85].

### 2.9. Selenium

Selenium is essential for the function of many antioxidant enzymes and offers protective effects from UV radiation-induced damage. Supplementation of selenium has been shown to be effective in patients with psoriasis while deficiency has been linked with epidermolysis bullosa and skin carcinogenesis [48]. Inversely, its presence may be linked to decreased skin cancer, particularly basal cell carcinoma (BCC) and squamous cell carcinoma (SCC) [46]. A food-derived selenium-rich extract was shown to be effective at decreasing wrinkles and aging with the promotion of collagen and fibroblasts [86]. Its bioavailability has been shown to be increased by Vitamin C [49].

### 2.10. Silicon

Silicon is a mineral that is consumed and then incorporated into orthosilicic acid (OSA), where it induces collagen secretion by fibroblasts and increases the production of glycosaminoglycans and elastin [24]. One study found that 600 mg/d of silicon supplementation not only helped with skin texture and appearance but also those of hair and nails that rely on the same proteins as the skin; in other studies, these results have been found with as little as 10 mg/day of silicon supplementation [24].

Many other compounds from whole foods probably contribute to skin health, from peptides to fibers to unknown phytonutrients. Much more work is needed to provide a robust evidence base for nutritional dermatology; however, the current evidence provides a preliminary framework for a skin-healthy diet.

## 3. Translating from Research to Dietary Pattern

Based on the literature discussed, a rudimentary scoring system is proposed for choosing a skin-healthy diet, see Table 1. This dietary pattern resembles other healthy dietary patterns such as the Mediterranean, DASH [87], and the Healthy Eating Index [88]. It also considers the literature reviewed here, showing how specific phytonutrients and nutrients benefit skin health. The maximum score is five for achieving the target for each diet factor. A score of three is provided for achieving 51–75% of the target, a score of one for 25–50%, and a score of zero for < 25% of the target. Even if the target is one serving, this allows an individual to have a half serving or less. This skin-healthy diet score allows individuals to assess their diets for the necessary nutrients and phytonutrients to maintain healthy skin. A skin-healthy dietary pattern was created to help individuals make daily food choices, see Table 2. The skin-healthy dietary pattern and its scoring system need to be tested, refined, and validated, in future work.

**Figure 1:**
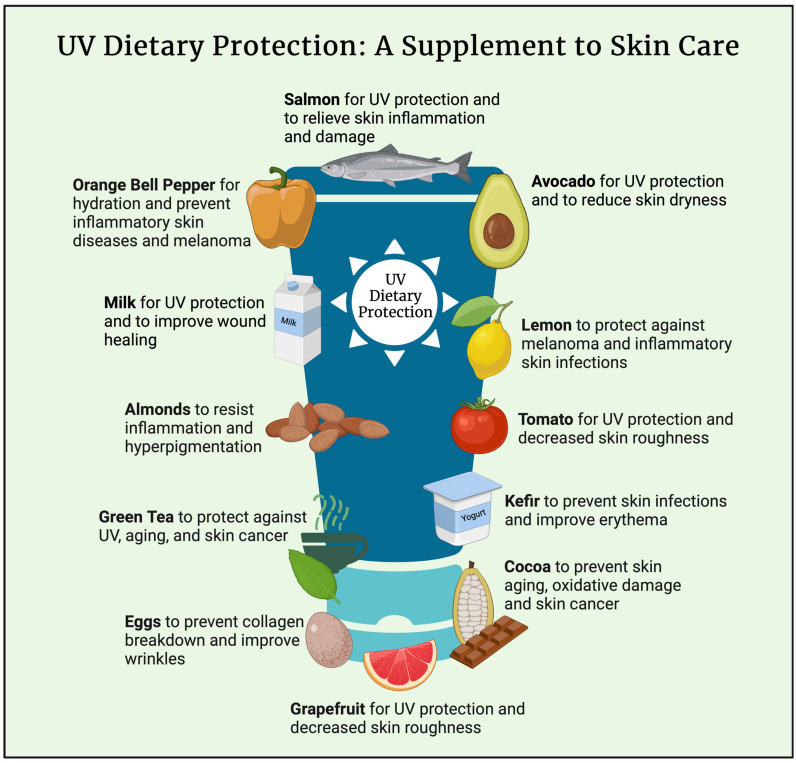
UV Dietary Protection: A Supplement to Skin Care; Created in BioRender. Assaf, S. (2024) https://BioRender.com/m40o422 (accessed on 10 November 2024).

An infographic was created (see Figure 1) from this dietary pattern to help visualize foods important for skin health. Each of these foods is high in several nutrients and phytonutrients that are involved in the pathophysiology of normal skin function and this infographic can easily be used in clinical settings to help educate patients on a skin-healthy diet, especially its importance in photoprotection, which may help reduce the risk of skin cancers. While diet is not a replacement for sunscreen or sunblock, the foods contain compounds that have been shown to be effective in reducing the risk of skin cancer. However, as sunscreen needs to be constantly reapplied throughout the day, dietary protection against UV damage may be a good adjunctive treatment to lower the risk of skin cancer. Although research into the gut–skin axis and nutritional dermatology is less common than other medical applications of nutrition, it is important for patients to know that nutrition is just as important for skin health. In combination, both the skin-healthy dietary pattern and infographic can be used clinically to provide further education and instruction on healthy eating with a focus on skin health.

## 4. Translating from Dietary Pattern to a Daily Diet

**Figure 2:**
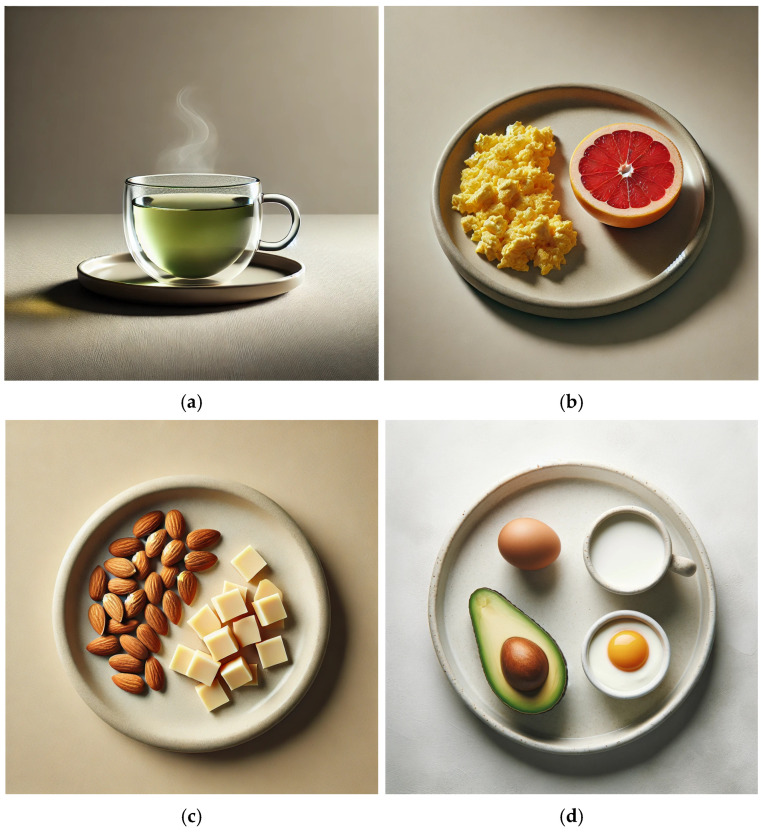

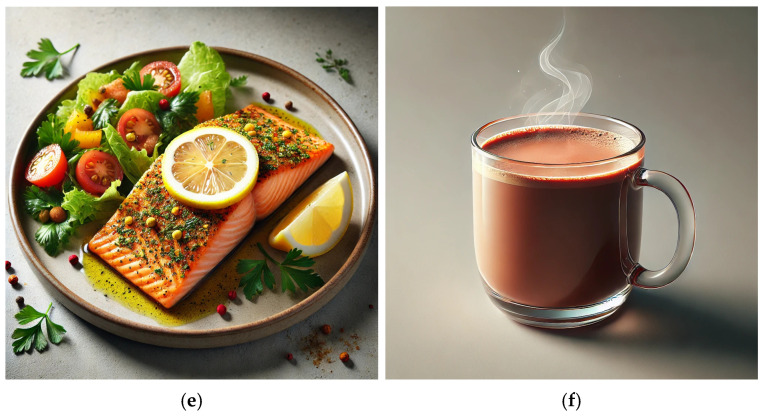
(**a**–**f**): A Skin-Healthy Sample Daily Diet (Visuals); Image Created using DALL-E (**a**) Generation ID: Mx76S2OyunYRaBZD, Seed Number: 2506571345; Description: A high-quality depiction of a hot cup of green tea served in a clear glass mug placed on a matching neutral-colored plate. The mug is clear without any unnatural reflections, and light steam rises delicately from the tea. The setting features a plain, neutral background with realistic textures and a serene minimalist presentation. (**b**) Generation ID: B1rJHKQYCFoV4F1T, Seed Number: 2768762300; Description: A high-quality depiction of scrambled eggs (fluffy and light) and half a freshly cut, vibrant grapefruit served on a simple, neutral-colored plate. The plate is set against a plain, neutral background with minimal shadows, emphasizing a serene and minimalist presentation. (**c**) Generation ID: 5COC10N1TTTQLne1, Seed Number: 1731434565; Description: A small neutral plate with almonds and cubed Gruyere cheese, arranged neatly in a minimalist style. (**d**) Generation ID: kRo5DZLSN3lfNrRx, Seed Number: 777524293; Description: A simple and clean breakfast arrangement. (**e**) Generation ID: cYzeygQGlGqi1foR, Seed Number: 1389760918; Description: A beautifully plated Fenugreek-rubbed Sockeye Salmon fillet, golden and slightly crisp, garnished with a lemon slice on top. Beside it, a vibrant salad featuring fresh parsley, diced tomatoes, crisp lettuce, and chunks of orange bell pepper, arranged on a neutral-colored plate with a neutral background. (**f**) Generation ID: k9gJmIGl2WDrkm7b, Seed: 3141118779; Description: A realistic image of hot chocolate in a clear mug, with steam rising from the top. The mug is placed on a lighter grey neutral background. The hot chocolate appears rich and creamy, with a frothy top. The clear mug shows the texture of the hot chocolate inside, and the scene is warmly lit to highlight the inviting nature of the drink.

A skin-focused diet was created that highlighted foods containing evidence-based compounds shown to have positive effects on skin health, see Table 2 for full descriptions. To formulate a sample diet that achieved all the recommended calories, minerals, vitamins, and phytonutrients possible, USDA FoodData Central (https://fdc.nal.usda.gov/, accessed on 10 November 2024), a public database of macro- and micro-nutrients, was used to search for the most nutrient-dense foods. FooDB (https://foodb.ca/, accessed on 10 November 2024) reports on the concentrations of phytonutrients such as polyphenols and was used to select phytonutrient-rich foods known to benefit skin health. The sample diet achieves the Daily Values for vitamins and minerals for adults and children aged four and up, see Table 3. Daily Values were chosen as they are already used on food labels and provide one value; the Daily Values are listed in Supplemental Table S1 (https://ods.od.nih.gov/HealthInformation/nutrientrecommendations.aspx, accessed on 10 November 2024). DALL-E (https://openart.ai/, accessed on 10 November 2024), an AI image generator, was used to model the meals and drinks using the proposed combination of foods and is shown in Figure 2a–f. The nutrients from the sample daily diet are shown in Table 3. Using FoodData Central and FooDB, the phytonutrients and other compounds from the sample daily diets are shown in Table 4.

## 5. Discussion

There is a wealth of data related to the effects of various nutrients and phytonutrients on various aspects of skin health, as discussed above. This then provides preliminary evidence-based, skin-healthy foods. However, this is the first time, to our knowledge, that the information has been combined to provide a skin-healthy diet, in addition to a scoring system and a sample daily diet. The skin-healthy diet and the sample daily diet are translations from the literature-related skin health benefits for key phytonutrients and nutrients. However, other important factors should be considered when personalizing any diet such as allergens, dietary preferences, cost, and cultural practices. For this reason, Table 1 and Figure 1 provide more general suggestions—a starting point—for dietary choices with a focus on skin health. As the evidence suggests that skin health requires dietary components beyond the essential nutrients, the focus of a skin-healthy diet should emphasize whole foods and limit ultra-processed foods (UPFs). Supplements can be considered; however, daily intakes at physiological levels may be the best strategy. It is also important to consider the appropriate preparation and cooking of polyphenol-rich foods, as there may be some loss of phytonutrients when foods are heated. However, addressing this aspect and the number of active metabolites created from phytonutrients are beyond the scope of this manuscript. In addition, it is important to recognize that the values represent an average value for nutrients or phytonutrients, and concentration varies greatly by region, season, processing, and storage.

The sample daily food met or exceeded the Daily Values (2000 kcal/d) for nutrients while also containing the phytonutrients shown to benefit skin health. Therefore, nutrient-dense foods were chosen to ensure that all foods add as many vitamins and minerals as possible. Certain vitamins and minerals exceed the Daily Value but are below the Tolerable Upper Intake Level, while others such as copper, molybdenum, chromium, and vitamin K do not have upper limits established. The Tolerable Upper Intake Levels are listed on the National Institutes of Health Office of Dietary Supplements website, available: https://ods.od.nih.gov/HealthInformation/nutrientrecommendations.aspx. In addition, for harm to occur intakes typically need to be above the Tolerable Upper Intake Level and be chronic intakes [98,99]. As dietary choices change each day, it is not expected that an individual would have the same foods each day.

Table 3 and Table 4 show each food’s nutrient and phytonutrient content, respectively. For example, green tea has numerous polyphenols and flavonoids (see Table 4). The grapefruit was selected because it is rich in β-carotene and contains lycopene due to its color. Grapefruit consumption is associated with higher vitamin C intake as well as an improved dietary pattern [100]. Orange bell pepper alone achieves the Daily Values for vitamin C (Table 3). Tomato was chosen as it is a good source of lycopene and β-carotene (Table 4). The herb parsley was chosen because it is a rich source of silicon and polyphenols and has one of the highest carotenoid concentrations among herbs [51]. Lemon was chosen as a rich source of vitamin C and selenium, but also contained various polyphenols (Table 3 and Table 4). Avocado was selected for its ω-3 and ω-6 fatty acids and is rich in vitamin K (Table 3 and Table 4). High-flavanol cocoa consumption was associated with improved skin health [70], thus a hot chocolate drink containing 200 g milk and 100 g cocoa was included in the daily diet; dark chocolate may also be an option. Milk is incredibly nutrient-dense, containing many vitamins and minerals, especially calcium, as well as zinc, selenium, and copper, which are important for skin health. The only sources of EPA and DHA in the daily diet were milk, eggs, kefir, and sockeye salmon. Eggs and sockeye salmon also contributed choline. Similarly, kefir was chosen as a low-calorie animal food that provides vitamin A and selenium. Fenugreek was primarily chosen as a rich source of choline, and fenugreek extract (fenugreek soaked in ethanol solvent) has also been shown to be effective in reducing collagen degradation and improving its production [101]. However, fenugreek is also a source of vitamin A, selenium, and silicon.

Collagen is essential to the composition of the dermis, and many foods contain the amino acids necessary for its production in the body; however, it can also be obtained in a supplement form [102]. Indeed, orally administered collagen was found to cause a statistically significant improvement in both skin elasticity and hydration [103]. However, collagen can also be obtained in whole foods, such as fish; thus, salmon provides further benefit in this skin-focused diet [104].

## 6. Conclusions

Optimizing human nutrition for skin health, and for overall health, requires whole foods with their complex milieu of phytonutrients, beyond the essential nutrients [105]. Macro- and micro-nutrients and phytonutrients all work together to produce collagen, elastin, and other proteins needed to repair and maintain healthy skin. Antioxidant and anti-inflammatory compounds play a role in blocking harmful UV rays or ensuring that their damage is minimized. Although topical creams and other products may protect the skin from the outside, it is essential to protect it from the inside. Supplements are commonly used to address deficiencies, but randomized trials have shown them to be less useful for the long-term reduction of disease risk compared with nutritional intake from a whole food diet [106]. A skin-healthy diet containing whole foods is proposed here that will not only meet macro- and micro-nutrient requirements, but also contain the phytonutrients shown to have a positive effect on skin health. The study of nutrition’s role in skin health and disease, or nutridermatology, has gained some traction in the past few years, but there is a greater need for research into the foods that we eat and their impact on the skin. There has been a large focus on sunscreen, skin beauty products, and even the addition of specific foods to a person’s normal diet, but more research is needed on the impact of a skin-healthy diet on various short- and long-term skin health outcomes. Future work should include developing more interest in food as medicine for dermatology and refining the preliminary skin-healthy dietary pattern. Perhaps with favorable results more people will eat for their skin care regimen rather than simply applying it.

## Figures and Tables

**Table 1:** Proposed Skin-Healthy Diet Pattern and Score.

Component	Target Intake	Scoring (Percent of Target)			
		75–100%	51–75%	25–50%	0–25%
Minimally processed (MP) food intake ^a^	≥50% of food consumed	5	3	1	0
Ultraprocessed food (UPF) intake ^b^	≤40% of food consumed	5	3	1	0
Total whole fruits ^c^	≥5 servings	5	3	1	0
Citrus fruits ^c^	≥2 servings	5	3	1	0
Total vegetables ^c^	≥3 servings	5	3	1	0
Green leafy vegetables ^c^	≥1 serving	5	3	1	0
Dairy ^d^	≥1 cup	5	3	1	0
Animal and plant proteins ^c^	≥5 servings	5	3	1	0
Seafood	≥1 serving	5	3	1	0
Eggs	≥2 servings	5	3	1	0
Refined Grains ^e^	≤20% of carbohydrate	5	3	1	0
Nuts and seeds ^c^	≥2 servings	5	3	1	0
Fatty acids ^f^					
Essential fatty acids	≥10% of energy				
LA	≥5% of energy	5	3	1	0
αLA	≥5% of energy	5	3	1	0
EPA + DHA	≥5% of energy	5	3	1	0
Sodium ^g^	≤2 g	5	3	1	0
Added Sugars ^h^	<5% of energy	5	3	1	0

Footnotes: ^a^ UPF consumption in US adults is up to 57% of energy [89]; ^b^ MP consumption is as low as 27% of energy [89]; ^c^ Dietary guidelines recommend an increase consumption of plant foods, and whole food plant-based diets are associated with better skin health [90,91]; ^d^ Dietary guidelines recommend a serving of dairy as part of a healthy diet; ^e^ Dietary guidelines recommend consuming more whole grains, at least 50%, and whole grains contribute to skin health [90]; ^f^ Essential fatty acids and ω-3 PUFA are beneficial for skin [58,92,93]; ^g^ Excess sodium may be stored in skin and contribute to aging of skin [94,95]; ^h^ Too much sugar may contribute to inflammation in skin [96,97].

**Table 2:** A sample of daily skin-healthy diet.

Sample Skin-Healthy Diet	Amount
Breakfast: Green Tea, Grapefruit, Scrambled Eggs cooked in Olive Oil	2.5 g Green Tea in Hot Water, 100 g Grapefruit, 150 g Eggs (~3) scrambled in 13.5 g (1 tbsp) Olive Oil
Snack: Almonds and Gruyère Cheese	50 g Almonds and 28 g (1 oz) Gruyère Cheese
Lunch: Boiled egg, ½ Avocado, Cup of Kefir	150 g Eggs (1 boiled), 50 g of Avocado, 100 g Kefir
Dinner: Fenugreek-rubbed Sockeye Salmon cooked in olive oil with salad of Parsley, Tomato, Lettuce, Orange Bell Pepper and dressed with Lemon juice	200 g Sockeye Salmon, 13.5 g Olive Oil, 12.5 g Fenugreek, 50 g Avocado, 100 g Orange bell pepper, 100 g Tomato, 100 g Lemon, 100 g Parsley, 100 g Lettuce
Snack 2: Hot Chocolate	100 g of Cocoa Powder in 200 g of Cow Milk
	Total Carbohydrates: 141.761 g Total Protein: 138.105 g Total Fat: 152.87 g Total Calories: 1975 (approximate)

**Table 3:** Nutrient Daily Values of the sample daily skin-healthy diet.

Vitamin/Mineral	RDI (4 and Up)	Total via Suggested Diet Using USDA FoodData Central (Ordered Highest to Lowest Content)	Total per Diet
Vitamin A (Retinol)	900 μg	421 μg (Parsley), 358 μg (Eggs), 171 μg (Kefir), 98 μg (Sockeye Salmon), 75.9 μg (Gruyère cheese), 62 μg (Milk), 58 μg (Grapefruit), 24 μg (Tomato), 7 μg (Avocado), 1 μg (Lemon), 0.375 μg (Fenugreek)	1276.275 μg
Vitamin C (Ascorbic Acid)	90 mg	158 mg (Orange Bell Pepper), 133 mg (Parsley), 53 mg (Lemon), 31.3 mg (Grapefruit), 17.8 mg (Tomato), 8.8 mg (Avocado), 0.375 mg (Fenugreek), 0.2 mg (Kefir)	402.475 mg
Calcium	1300 mg	283 mg (Gruyère Cheese), 246 mg (Milk), 135 mg (Almonds), 138 mg (Parsley), 130 mg (Kefir), 128 mg (Cocoa Powder), 96 mg (Eggs), 28 mg (Lettuce), 26 mg (Lemon), 22 mg (Grapefruit), 22 mg (Fenugreek), 18 mg (Sockeye Salmon), 13 mg (Avocado), 10 mg (Tomato), 5 mg (Orange Bell Pepper), 0.27 mg (Olive Oil)	1300.27 mg
Iron	18 mg	13.9 mg (Cocoa powder), 4.1875 mg (Fenugreek), 3.34 mg (Eggs), 6.2 mg (Parsley), 1.855 mg (Almonds), 0.86 mg (Sockeye Salmon), 0.61 mg (Avocado), 0.6 mg (Lemon), 0.37 mg (Orange Bell Pepper), 0.27 mg (Lettuce), 0.152 mg (Olive Oil), 0.1 mg (Tomato), 0.08 mg (Grapefruit), 0.04 mg (Kefir), 0.048 mg (Gruyère Cheese)	32.6125 mg
Vitamin D (Calciferol)	20 μg	28.2 μg (Sockeye Salmon), 4.92 μg (Eggs), 1.92 μg (Milk), 0.168 μg (Gruyère Cheese)	35.208 μg
Vitamin E (Tocopherol)	15 mg	12.8 mg (Almonds), 3.88 mg (Olive Oil), 1.66 mg (Sockeye Salmon), 0.75 mg (Parsley), 0.15 mg (Lemon), 0.13 mg (Grapefruit), 0.1 mg (Milk), 0.1 mg (Cocoa powder), 0.078 mg (Gruyère Cheese), 0.02 mg (Kefir)	19.668 mg
Vitamin K	120 μg	1640 μg (Parsley), 83.4 μg (Lettuce), 21 μg (Avocado), 16.26 μg (Olive Oil), 2.500 μg (Cocoa Powder), 0.756 μg (Gruyère Cheese) 0.4 μg (Sockeye Salmon)	1764.316 μg
Thiamin (Vitamin B1)	1.2 mg	0.264 mg (Sockeye Salmon), 0.154 mg (Eggs), 0.112 mg (Milk), 0.1025 mg (Almonds), 0.086 mg (Parsley), 0.075 mg (Avocado), 0.063 mg (Lettuce), 0.056 mg (Tomato), 0.055 mg (Orange Bell Pepper), 0.043 mg (Grapefruit), 0.04025 mg (Fenugreek), 0.04 mg (Lemon), 0.030 mg (Kefir), 0.017 mg (Gruyère Cheese)	1.21575 mg
Riboflavin (Vitamin B2)	1.3 mg	0.838 mg (Egg), 0.414 mg (Sockeye Salmon), 0.57 mg (Almonds), 0.276 mg (Milk), 0.241 mg (Cocoa Powder), 0.143 mg (Avocado), 0.135 mg (Kefir), 0.102 mg (Orange Bell Pepper), 0.098 mg (Parsley), 0.078 mg (Gruyère Cheese), 0.04575 mg (Fenugreek), 0.032 mg (Grapefruit), 0.02 mg (Lemon), 0.1 mg (Tomato)	3.09275 mg
Niacin (Vitamin B3)	16 mg	17.02 mg (Sockeye Salmon), 2.18 mg (Cocoa Powder), 1.91 mg (Avocado), 1.81 mg (Almonds), 1.31 mg (Parsley), 1.08 mg (Orange Bell Pepper), 0.533 mg (Tomato), 0.4 mg (Eggs), 0.371 mg (Lettuce), 0.214475 mg (Green Tea), 0.205 mg (Fenugreek), 0.204 mg (Grapefruit), 0.21 mg (Milk), 0.15 mg (Kefir), 0.1 mg (Lemon), 0.03 mg (Gruyère Cheese)	27.627 mg
Pyridoxine (Vitamin B6)	1.6 mg	1.462 mg (Sockeye Salmon), 0.332 mg (Orange Bell Pepper), 0.287 mg (Avocado), 0.126 mg (Eggs), 0.122 mg (Milk), 0.118 mg (Cocoa Powder), 0.09 mg (Parsley), 0.079 mg (Tomato), 0.075 mg (Fenugreek), 0.063 mg (Lettuce), 0.058 mg (Kefir), 0.053 mg (Grapefruit), 0.0685 mg (Almonds), 0.023 mg (Gruyère Cheese)	3.0365 mg
Folate/Folic Acid	400 μg	152 μg (Parsley), 142 μg (Eggs), 89 μg (Avocado), 32 μg (Cocoa Powder), 22 μg (Almonds), 13 μg (Grapefruit), 13 μg (Kefir), 12 μg (Sockeye Salmon), 11 μg (Lemon), 10 μg (Tomato), 7.125 μg (Fenugreek), 2.8 μg (Gruyère Cheese)	505.925 μg
Vitamin B12 (Cobalamin)	2.4 μg	9.38 μg (Sockeye Salmon), 2.4 μg (Eggs), 1.08 μg (Milk), 0.448 μg (Gruyère Cheese), 0.29 μg (Kefir)	13.598 μg
Biotin	30 μg	45.9 μg (Eggs), 0.58 μg (Orange Bell Pepper), 0.469 μg (Tomato)	46.949 μg
Pantothenic acid (Vitamin B5)	5 mg	2.14 mg (Sockeye Salmon), 1.46 mg (Avocado), 0.724 mg (Milk), 0.385 mg (Kefir), 0.262 mg (Grapefruit), 0.254 mg (Cocoa Powder), 0.23505 mg (Almonds), 0.4 mg (Parsley), 0.19 mg (Lemon), 0.157 mg (Gruyère Cheese)	6.20705 mg
Phosphorus	1250 mg	734 mg (Cocoa Powder), 514 mg (Sockeye Salmon), 368 mg (Eggs), 240.5 mg (Almonds), 202 mg (Milk), 105 mg (Kefir), 58 mg/100 g (Parsley), 54 mg/100 g (Avocado), 37 mg (Fenugreek), 27 mg (Orange Bell Pepper), 23 mg (Lettuce), 19 mg (Tomato), 18 mg (Grapefruit), 16 mg (Lemon), 169 mg (Gruyère Cheese)	2584.5 mg
Iodine	150 μg	98.2 μg (Eggs), 75.8 μg (Milk), 36.8 μg (Sockeye Salmon)	210.8 μg
Magnesium	420 mg	499 mg (Cocoa Powder), 135 mg (Almonds), 60 mg (Sockeye Salmon), 50 mg (Parsley), 29 mg (Avocado), 23.875 mg (Fenugreek), 23.8 mg (Milk), 12 mg (Kefir), 12 mg (Lettuce), 10.4 mg (Orange Bell Pepper), 10.1 mg (Gruyère Cheese), 9 mg (Grapefruit), 8.1 mg (Tomato), 8 mg (Lemon)	913.075 mg
Zinc	11 mg	6.81 mg (Cocoa powder), 2.48 mg (Eggs), 1.56 mg (Almonds), 1.09 mg (Gruyère Cheese), 0.92 mg (Sockeye Salmon), 0.84 mg (Milk), 1.07 mg (Parsley), 0.68 mg (Avocado), 0.46 mg (Kefir), 0.3125 mg (Fenugreek), 0.25 mg (Lettuce), 0.24 mg (Orange Bell Pepper), 0.07 mg (Grapefruit), 0.08 mg (Tomato), 0.06 mg (Lemon)	16.9225 mg
Selenium	55 μg	62.2 μg (Egg), 59.6 μg (Sockeye Salmon), 14.3 μg (Cocoa Powder), 3.8 μg (Milk), 3.6 μg (Kefir), 4.06 μg (Gruyère Cheese), 2.5 μg (Tomato), 2.06 μg (Almonds), 0.7875 μg (Fenugreek), 0.4 μg (Lemon), 0.4 μg (Avocado), 0.1 μg (Parsley), 0.1 μg (Grapefruit)	154.2075 μg
Copper	0.9 mg	3.79 mg (Cocoa Powder), 0.515 mg (Almonds), 0.2 mg (Eggs), 0.149 mg (Parsley), 0.13875 mg (Fenugreek), 0.128 mg (Sockeye Salmon), 0.17 mg (Avocado), 0.037 mg (Lemon), 0.035 mg (Orange Bell Pepper), 0.032 mg (Tomato), 0.032 mg (Grapefruit), 0.049 mg (Lettuce), 0.009 mg (Kefir), 0.009 mg (Gruyère Cheese), 0.002 mg (Milk)	5.29575 mg
Manganese	2.3 mg	3.84 mg (Cocoa Powder), 1.06 mg (Almonds), 0.227 mg (Lettuce), 0.15375 mg (Fenugreek), 0.149 mg (Orange Bell Pepper), 0.149 mg (Avocado), 0.16 mg (Parsley), 0.1 mg (Eggs), 0.087 mg (Tomato), 0.05 mg (Lemon), 0.022 mg (Sockeye Salmon), 0.022 mg (Grapefruit), 0.005 mg (Kefir), 0.005 mg (Gruyère Cheese)	6.01435 mg
Chromium	35 μg	173.00 μg (Cocoa Powder), 50 μg (Fenugreek), 15.93 μg (Parsley), 8.375 μg (Green Tea), 0.13 μg (Lemon)	247.435 μg
Molybdenum	45 μg	266 μg (Milk), 30 μg/50 g (Almonds), 0.8 μg (Lettuce)	296.8 μg
Chloride	2300 mg	1045.462 mg (Egg), 301 mg (Sockeye Salmon), 285.923 mg (Tomato), 278.04 mg (Lettuce), 276.918 mg (Milk), 106.525 mg (Parsley), 34.970875 mg (Fenugreek), 19.429 mg (Cocoa Powder), 13.667 mg (Avocado), 12.800 mg (Lemon), 10.092 mg (Orange Bell Pepper), 1.000 mg (Kefir)	2385.827 mg
Potassium	4700 mg	1520 mg (Cocoa powder), 734 mg (Sockeye Salmon), 554 mg (Parsley), 507 mg (Avocado), 366.5 mg (Almonds), 300 mg (Milk), 260 mg (Lettuce), 201 mg (Orange Bell Pepper), 193 mg (Tomato), 167 mg (Kefir), 138 mg (Lemon), 135.000 mg (Grapefruit), 96.25 mg (Fenugreek), 22.7 mg (Gruyère Cheese), 0.27 mg (Olive Oil)	5520.82 mg
Choline	550 mg	670 mg (Egg), 189.2 mg (Sockeye Salmon), 35.6 mg (Milk), 21.05 mg (Almonds), 15.2 mg (Kefir), 14.2 mg (Avocado), 12.800 mg (Parsley), 12 mg (Cocoa powder), 7.700 mg (Grapefruit), 5.100 mg (Lemon), 4.31 mg (Gruyère Cheese), 0.082 mg (Olive Oil)	987.242 mg
Sodium		258 mg (Eggs), 200 mg (Gruyère Cheese), 156 mg (Sockeye Salmon), 76 mg (Milk), 56 mg (Parsley), 40 mg (Kefir), 23 mg (Lettuce), 21 mg (Cocoa), 8.375 mg (Fenugreek), 8 mg (Avocado), 2.5 mg (Tomato), 2.4 mg (Orange Bell Pepper), 2 mg (Lemon), 0.54 mg (Olive Oil), 0.5 mg (Almonds)	854.315 mg

**Table 4:** Phytonutrients contained in the sample daily skin-healthy diet.

Compound	Total via Suggested Diet Using Foodb	Total per Diet
Beta carotene (Carotenoid)	Orange Bell Pepper (964.50 μg) Grapefruit (686 μg) Tomato (276 μg) Avocado (63 μg) Milk (14 μg) Lemon (3 μg) Almonds (0.5 μg)	2070 μg
Lycopene (Carotenoid)	Tomato (2860 μg) Grapefruit (1420 μg)	1826 mg
Astaxanthin (Carotenoid)	N/A	N/A
ALA—Alpha-linolenic acid (ω-3 FA)	Avocado (111 mg) Egg (72 mg) Milk (24 mg) Kefir (6 mg) Almonds (1.5 mg)	214.5 mg
DHA—Docosahexaenoic acid (ω-3 FA)	Sockeye salmon (942 mg) Eggs (116 mg)	1058 mg
EPA—Eicosapentaenoic acid (ω-3 FA)	Sockeye salmon (502 mg) Milk (2 mg)	504 mg
LA—Linoleic acid (ω-6 FA)	Almonds (6.15 g) Avocado (1.67 g) Olive Oil (1.32 g) Gruyère Cheese (0.364 g) Sockeye Salmon (0.32 g) Milk (0.194 g)	8.698 mg
GLA—γ-linolenic acid (ω- 6 FA)	Olive Oil (206 mg) Gruyère Cheese (121 mg) Sockeye Salmon (96 mg) Milk (54 mg) Kefir (6 mg) Almonds (1.5 mg)	279.5 mg
AA—Arachidonic acid (ω- 6 FA)	Milk (8 mg)	8 mg
Silicon	Parsley (74.250 mg) Almonds (48 mg) Orange bell pepper (1.700 mg) Fenugreek (0.5875 mg)	124.5375 mg
Polyphenols	Parsley (4,731.633 mg) [Quercetin 3-rutinoside (3000.000 mg) Apigenin (1612.081 mg) Isorhamnetin (110.425 mg) Luteolin (7.350 mg) Myricetin (1.347 mg) Quercetin (0.28333 mg) Kaempferol (0.14667 mg)] Avocado (4,700.72 mg) [Chinese Tannin (4700.000 mg) alpha-Catechin (0.37000 mg) Cyanidin (0.33000 mg) Procyanidin B2 (0.02000 mg)] Fenugreek (3,500.214 mg) [Lignin (3500.000 mg) Quercetin 3-rutinoside (0.11659375 mg) Quercetin (0.09774375 mg)] Green Tea (692.469 mg) [Chinese tannin (379.75 mg) ent-Gallocatechin 3-gallate (102.25 mg) Epigallocatechin (16.1136 mg) (R)-Oxypeucedanin (15.75 mg), Epicatechin 3-gallate (15.609175 mg) Theaflavin (105.23625 mg) Quercetin 3-rutinoside (15.19325 mg) Epicatechin 3-(3-methylgallate) (12.6125 mg) Epigallocatechin 3-(3-methylgallate) (4.925 mg) Epigallocatechin 3-p-coumarate (2.075 mg) Theasinensin B (2.05 mg) Gallocatechin (2.034 mg) Strictinin (1.825 mg) Assamicain A (1.45 mg) Theogallin (1.3265 mg) (-)-Epiafzelechin 3-gallate (0.925 mg) Assamicain C (0.85 mg) Epigallocatechin-(4beta- > 8)-epicatechin 3’-gallate (0.7 mg) Catechin 3-gallate (0.592 mg) Gallocatechin-(4alpha- > 8)-epicatechin (0.5625 mg) Procyanidin B1 (0.53325 mg) Epicatechin-(4beta- > 8)-epigallocatechin 3’-gallate (0.525 mg) Theaflavin 3’-gallate (0.5095 mg) Theasinensin F (0.5 mg) 6,8-Di-C-beta-D-arabinopyranosylapigenin (0.5 mg) Procyanidin B4 (0.45375 mg) Theasinensin D (0.45 mg) Theaflavin 3,3’-digallate (0.44025 mg) Procyanidin B2 (0.4085 mg) Theasinensin E (0.35 mg) alpha-Catechin (0.3275 mg) Epigallocatechin 3-cinnamate (0.325 mg) Catechin (0.30375 mg) Kaempferol 3-rutinoside (0.2845 mg) Astragalin (0.276 mg) Quercetin 3-O-glucosyl-rhamnosyl-glucoside (0.2755 mg) Oolonghomobisflavan A (0.275 mg) 8-C-Ascorbylepigallocatechin 3-gallate (0.275 mg) Quercetin (0.2645 mg) Epigallocatechin 3,5-digallate (0.25 mg) Prodelphinidin B3 (0.24 mg) Procyanidin C1 (0.22875 mg) Hyperin (0.213935 mg) Theaflavin 3-gallate (0.19752 mg) 1,4,6-Trigalloyl-beta-D-glucopyranose (0.1875 mg) Kaempferol (0.1799025 mg) Oolonghomobisflavan B (0.175 mg) Quercetin 3-O-glucosyl-rhamnosyl-galactoside (0.168555 mg) Kaempferol 3-O-glucosyl-rhamnosyl-glucoside (0.1682125 mg) Isoquercitrin (0.163545 mg) (-)-Epiafzelechin (0.15 mg) Procyanidin B7 (0.13625 mg) Myricetin (0.11136 mg) Procyanidin B3 (0.1075 mg) Epicatechin-(4beta- > 8)-epigallocatechin 3,3’-digallate (0.1075 mg) Kaempferol 3-galactoside (0.0966075 mg) Gallocatechin 3-gallate (0.0962825 mg) Theaflagallin (0.0875 mg) Kaempferol 3-O-glucosyl-rhamnosyl-galactoside (0.07029 mg) Quercetin 3-O-rhamnosyl-galactoside (0.0595725 mg) Oolongtheanin (0.05 mg) Epigallocatechin 3-caffeate (0.05 mg) Degalloyl theasinensin F (0.0375 mg) Quercitrin (0.0155075 mg) Secoisolariciresinol (0.00767 mg) Avicularin (0.0049 mg) Procyanidin B5 (0.00125 mg)] Cocoa Powder (479.836 mg) [alpha-Catechin (126.515 mg) Procyanidin B1 (112.000 mg) Catechin (107.750 mg) Procyanidin B2 (71.571 mg) Cinnamtannin A2 (33.167 mg) Procyanidin C1 (23.833 mg) Epicatechin-(2alpha- > 7,4alpha- > 8)-epicatechin 3-galactoside (5.000 mg)] Lemon (77.139 mg) [Hesperidin (21.397 mg) Eriodictyol (19.480 mg) Hesperetin (17.100 mg) Eriodictyol 7-rutinoside (11.737 mg) Diosmetin 7-rutinoside (2.920 mg) Scolymoside (1.850 mg) Narirutin (0.64000 mg) Luteolin (0.63500 mg) (S)-Naringenin (0.55000 mg) Eriodictyol 7-neohesperidoside (0.31353 mg) Quercetin (0.28556 mg) Quercetin 3-rutinoside (0.15000 mg) Hesperetin 7-neohesperidoside (0.08056 mg)] Grapefruit (68.9 mg) [Naringin (45.050 mg) Narirutin (14.950 mg) Naringin 6”-malonate (2.267 mg) Naringenin 4’-glucoside (1.833 mg) Narirutin 4’-glucoside (1.233 mg) Rhoifolin (1.167 mg) Poncirin (0.91667 mg) Rhoifolin 4-glucoside (0.65000 mg) Hesperetin 7-neohesperidoside (0.61667 mg) Eriodictyol 7-neohesperidoside (0.21667 mg)] Lettuce (22.626 mg) [Quercetin (10.476 mg) Quercetin 3-(6”-malonyl-glucoside) (6.149 mg) Cyanidin 3-(6”-malonylglucoside) (1.459 mg) Luteolin 7-glucuronide (1.457 mg) Isoquercitrin (1.287 mg) Cyanidin (0.62800 mg) Quercitrin (0.49325 mg) Cyanidin 3-glucoside (0.31289 mg) Quercetin 3-O-(6”-malonyl-glucoside) 7-O-glucoside (0.19532 mg) Hyperin (0.12163 mg) Quercetin 3-rutinoside (0.02023 mg) Kaempferol (0.01372 mg) Myricetin (0.00665 mg) Luteolin (0.00609 mg) Apigenin (0.00009 mg)] Almonds (4.052 mg) [Epigallocatechin (1.3 mg) Catechin (0.6395 mg) Kaempferol 3-rutonside (0.516 mg) Hyperin (0.394735 mg) Isorhamnetin 3-galactoside (0.294135 mg) Gallocatechin 3-gallate (0.23 mg) Quercetin 3-rutinoside (0.129475 mg) Eriodictyol (0.127505 mg) Vanillic Acid (0.086 mg) Prunin (0.083645 mg) Isorhamnetin (0.07415 mg) 3,4’,5,6,7,8-Hexahydroxyflavone (0.05596 mg) (S)-Naringenin (0.0481 mg) Isoquercitrin (0.046855 mg) Astragalin (0.009825 mg) Quercetin (0.0067 mg) Kaempferol 3-galactoside (0.006545 mg) 4-Hydroxybenzoic acid (0.00205 mg) Kaempferol (0.00102 mg)] Orange bell pepper (1.2 mg) [Luteolin (1.100 mg) Myricetin (0.10000 mg)] Tomato (0.05447 mg) [Kaempferol (0.04000 mg) Quercetin (0.00642 mg) Isorhamnetin (0.00530 mg) Quercetin 3-rutinoside (0.00160 mg) Myricetin (0.00115 mg)]	14,278.84 mg

## Data Availability

The original data presented in this manuscript are openly available in USDA FoodData Central [https://fdc.nal.usda.gov/ (accessed on 12 November 2024)] and FooDB [https://foodb.ca/ (accessed on 12 November 2024)].

## Supplementary Materials

The following supporting information can be downloaded at: https://www.mdpi.com/article/10.3390/nu17010060/s1, Table S1: U.S. Food label Daily Values, Daily Reference Values (DRV) and Reference Daily Intakes (RDI).
