# Supplementary material for: Nutritional Dermatology: Optimizing Dietary Choices for Skin Health
Source: Nutrients. 2024 Dec 27;17(1):60. doi: 10.3390/nu17010060 (PMC11723311; doi:10.3390/nu17010060)
Supplement: Supplementary file 1 [file nutrients-17-00060-s001.zip › nutrients-3361660-supplementary.pdf]

Supplementary Table S1. U.S. Food label Daily Values, Daily Reference Values (DRV) and Reference Daily Intakes (RDI)

Nutrient		Unit	Value
**DRV**			
	Fat	g	78
	Saturated fat	g	20
	Cholesterol	mg	300
	Carbohydrate	g	275
	Sodium	mg	2,300
	Fiber	g	28
	Protein	g	50
	Added sugars	g	50
**RDI**			
	Vitamin A	μg RAE	900
	Vitamin C	mg	90
	Calcium	mg	1,300
	Iron	mg	18
	Vitamin D	μg	20
	Vitamin E	mg	15
	Vitamin K	μg	120
	Thiamin	mg	1.2
	Riboflavin	mg	1.3
	Niacin	mg NE	16
	Vitamin B6	mg	1.7
	Folate	μg DFE	400
	Vitamin B12	μg	2.4
	Biotin	μg	30
	Pantothenic acid	mg	5
	Phosphorus	mg	1,250
	Iodine	μg	150
	Magnesium	mg	420
	Zinc	mg	11
	Selenium	μg	55
	Copper	mg	0.9
	Manganese	mg	2.3
	Chromium	μg	35
	Molybdenum	μg	45
	Chloride	mg	2,300
	Potassium	mg	4,700
	Choline	mg	550

RAE = Retinol Activity Equivalents; 1 μg RAE=1 μg retinol, 2 μg supplemental β-carotene, 12 μg β-carotene, or 24 μg α-carotene, or 24 μg β-cryptoxanthin

IU = International Units

NE = Niacin Equivalents, 1 mg NE = 1 mg niacin = 60 mg tryptophan

DFE = Dietary Folate Equivalents; 1 DFE = 1 μg naturally occurring folate = 0.6 μg folic acid

Available here: https://ods.od.nih.gov/HealthInformation/nutrientrecommendations.aspx
